# Sarcoidosis in Native and Transplanted Kidneys: Incidence, Pathologic Findings, and Clinical Course

**DOI:** 10.1371/journal.pone.0110778

**Published:** 2014-10-20

**Authors:** Serena M. Bagnasco, Srinivas Gottipati, Edward Kraus, Nada Alachkar, Robert A. Montgomery, Lorraine C. Racusen, Lois J. Arend

**Affiliations:** 1 Department of Pathology, Johns Hopkins University, Baltimore, Maryland, United States of America; 2 Department of Medicine, Johns Hopkins University, Baltimore, Maryland, United States of America; 3 Department of Surgery, Johns Hopkins University, Baltimore, Maryland, United States of America; University of Torino, Italy

## Abstract

Renal involvement by sarcoidosis in native and transplanted kidneys classically presents as non caseating granulomatous interstitial nephritis. However, the incidence of sarcoidosis in native and transplant kidney biopsies, its frequency as a cause of end stage renal disease and its recurrence in renal allograft are not well defined, which prompted this study. The electronic medical records and the pathology findings in native and transplant kidney biopsies reviewed at the Johns Hopkins Hospital from 1/1/2000 to 6/30/2011 were searched. A total of 51 patients with a diagnosis of sarcoidosis and renal abnormalities requiring a native kidney biopsy were identified. Granulomatous interstitial nephritis, consistent with renal sarcoidosis was identified in kidney biopsies from 19 of these subjects (37%). This is equivalent to a frequency of 0.18% of this diagnosis in a total of 10,023 biopsies from native kidney reviewed at our institution. Follow-up information was available in 10 patients with biopsy-proven renal sarcoidosis: 6 responded to treatment with prednisone, one progressed to end stage renal disease. Renal sarcoidosis was the primary cause of end stage renal disease in only 2 out of 2,331 transplants performed. Only one biopsy-proven recurrence of sarcoidosis granulomatous interstitial nephritis was identified.

**Conclusions:**

Renal involvement by sarcoidosis in the form of granulomatous interstitial nephritis was a rare finding in biopsies from native kidneys reviewed at our center, and was found to be a rare cause of end stage renal disease. However, our observations indicate that recurrence of sarcoid granulomatous inflammation may occur in the transplanted kidney of patients with sarcoidosis as the original kidney disease.

## Introduction

Sarcoidosis is a systemic disorder of unclear etiology, which results from an abnormal cell-mediated immune reaction, and is characterized by non caseating granulomatous inflammation with epithelioid cells and multinucleated giant cells. Sarcoidosis affects individuals mostly in their third or fourth decades. Its incidence has been reported to be as high as 40 cases per 100,000 in Europe [1]. In the US population sarcoidosis is about three times more common in blacks than in white [2].

Sarcoidosis most commonly affects the lungs, but multiple organs such as the central nervous system, liver, heart, skin, and kidney can be involved.

Sarcoid involvement of the native kidney, in the form of granulomatous tubulointerstitial nephritis, is unusual, but its frequency is unclear, with estimates up to 30% of patients with this disorder in earlier studies [3] and as low as 0.7% in more recent reports [4], [5]. It is also not clear how often renal sarcoidosis leads to end stage renal disease (ESRD).

Only a few cases of recurrent sarcoidosis with granulomatous inflammation in the transplanted kidney have been described [6], [7], [8], [9]. A multicenter study from France [10], reported recurrence in the transplanted kidney of 3 recipients with sarcoidosis as the original disease.

The main objectives of this study were to evaluate the incidence of renal sarcoidosis in patients with diagnosis of systemic sarcoidosis and with kidney abnormalities requiring a kidney biopsies, its frequency as a cause of ESRD in transplant recipients, the incidence of recurrence of renal sarcoidosis in transplanted kidneys, and the overall frequency of interstitial granulomatous sarcoidosis in native kidney biopsies. To this end we searched the electronic medical records and the archives of the Department of Pathology at the Johns Hopkins Hospital over a period between 2000 and 2011. Our findings are described and discussed here.

The study was approved by the John Hopkins Institutional Review Board (protocol NA_00001141, which includes approval by Johns Hopkins IRB of a waiver of consent for subjects whose clinical information was used for studies covered by this protocol).

## Methods

### Patients

Computerized records were searched to identify kidney biopsies reviewed in the Department of Pathology at Johns Hopkins University from 1/1/2000 to 6/30/2011.

The demographic and clinical information provided at time of biopsy and afterwards were recorded, when available, for subjects with native kidney biopsies and for those with renal graft biopsies, and included: age at biopsy, gender, race, relevant past medical history, serum creatinine (mg/dL), urinalysis, pertinent serology tests, clinical course and follow up (when documented).

GFR was estimated according to the MDRD calculation [11].

### Biopsies

Native kidney biopsies were processed for light microscopy, immunofluorescence and electron microscopy. Special stains of sections from paraffin embedded tissue such as Grocott methenamine silver, and stain for Acid Fast Bacilli were performed to rule out fungi and mycobacteria. Kidney allograft biopsies were stained for C4d as previously described [12], [13] and graded for rejection according to the Banff classification [14], [15].

Biopsies from patients with histologic findings of granulomatous inflammation were re-examined for this study by one nephropathologist (SMB).

### Statistical analysis

Continuous variables with normal distribution were analyzed by un-paired, two-tailed Student’s T test, with P≤0.05 as indicative of significant difference. Chi square test or Mann-Whitney test were used for categorical variables. Statistical analyses were performed with GraphPad Prism software, version 5 (GraphPad Software, Inc., San Diego, CA, USA).

## Results

A total of 14,306 kidney biopsies were reviewed in the Department of Pathology at the Johns Hopkins Hospital between January 2000 to June 2011, of which 10,023 were from native kidneys, and 4,283 from kidney allografts.

A diagnosis of sarcoidosis was reported in the medical history of 52 patients who had renal/urinary abnormalities requiring native or allograft kidney biopsy, with a total of 57 kidney biopsies performed on these subjects. The demographic data in Table 1 show that age ranged from 20 to 80 years at time of kidney biopsy, there was a higher proportion of females (57%), and a higher number of black individuals (73%).

**Table 1:** Characteristics of all patients with a diagnosis of sarcoidosis, in whom a biopsy from native or transplanted kidney was performed.

Patient number	52
Gender: Male/Female	22/30
Age at time of biopsy	50±13
Race: Black/White (N)	31/11 (42)
Hypertension	21
Diabetes	12

Values are presented as Mean ± SD. (N) indicates the number of patients for whom this information was available.

In 51 patients with a history of sarcoidosis a biopsy of the native kidney was performed, the pathologic findings in these biopsies are listed in Table 2.

**Table 2:** Main histological diagnoses in 56 native kidney biopsies from 51 patients carrying a diagnosis of sarcoidosis.

Diagnosis	N
Granulomatous interstitial nephritis*	19
Interstitial nephritis without granuloma	8
Diabetic nephropathy	7
Focal Segmental Glomerulosclerosis	6
Chronic/Advanced sclerosing changes**	6
Immune complex mediated glomerulonephritis	3
Acute tubular injury	3
Amyloid	1
Membranous glomerulopathy	1
Thin glomerular basement membrane disease	1
Non specific changes	1

*A diagnosis of granulomatous interstitial nephritis consistent with renal sarcoidosis was rendered when other potential differential diagnoses (drug reactions, bacterial, mycobacterial and fungal infections, *de novo* or recurrent Wegener’ granulomatosis, foreign body reaction) could be excluded.

**This category includes: Severe global glomerulosclerosis; Tubulointerstitial scarring; Hypertensive nephrosclerosis; Transplant glomerulopathy with tubulointerstitial scarring.

Interstitial nephritis was the main diagnosis in approximately half of these patients (27 cases, granulomatous and non granulomatous), the remaining patients showed renal lesions without no obvious association with sarcoidosis. Granulomatous interstitial nephritis as the manifestation of renal sarcoidosis was identified in 19 of these biopsies, in which infections, drug reactions, and Wegener’ granulomatosis could be ruled out. This is equivalent to a frequency of 0.18% of this diagnosis in a total of 10,023 biopsies from native kidney reviewed at our institution.

There were no distinct demographic or clinical features in those patients with granulomatous interstitial involvement by sarcoidosis, compared to those with a history of sarcoidosis but without renal granulomatous inflammation.

The 19 patients with a biopsy-proven granulomatous sarcoidosis in the native kidney had abnormal serum creatinine: 3.96±2.35 mg/dl (average ± SD; range 1.9–9 mg/dl). Proteinuria was known to be present in 12 patients, ranging from trace to 2.5 g/24 hours, it was quantified in 8 patients with an average of 0.94±0.65 g/24 hours. Hypercalcemia was present in 7 of 19 patients but only 3 had evidence of microcalcifications in the kidney biopsy.

**Figure 1:**
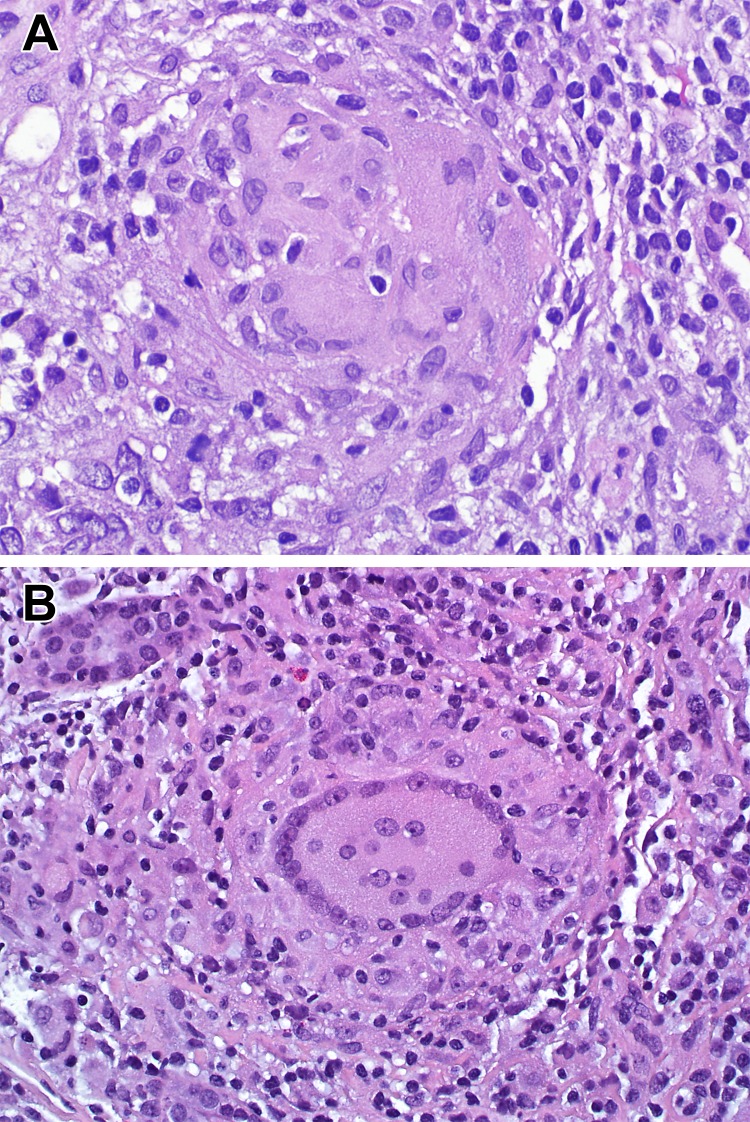
Moderate to severe interstitial inflammation with non caseating granuloma in renal sarcoidosis (A). Multinucleated cells in granulomatous inflammatory infiltrate in renal sarcoidosis (B).

Histologic evaluation of these 19 biopsies showed that the non caseating granulomatous inflammation was usually extensive, involving more than 50% of the kidney sample in 16 biopsies (figure 1). Most biopsies showed only rare eosinophils, and only 3 biopsies showed eosinophils present in more than one cluster with >4 eosinophils per high power field, usually embedded in the granulomatous inflammation. There was more than 50% global glomerulosclerosis in 7 biopsies. Tubulointerstitial scarring involving more than 50% of the sampled kidney was identified in 12 biopsies.

Only limited data on follow up were available in 10 patients with renal granulomatous sarcoidosis in the native kidney (Table 3). The patients with this lesion were treated with doses of prednisone ranging from 25 to 80 mg/day, tapered or suspended within one to three years, with general improvement of renal function, although in 4 patients with more than 50% tubulointerstitial scarring serum creatinine did not return to previous baseline level, and one patient progressed to ESRD requiring dialysis.

**Table 3:** Follow up summary for patients with renal granulomatous sarcoidosis in the native kidney.

Patients	10
Gender F/M	5/5
Race B/W	7/3
Age years	49±14 (50)
Serum Creatinine, mg/dl	
Baseline	1.6±0.5 (1.4)
At biopsy	4.3±2.6 (3.0)
At last FU	2.4±1.6 (1.8)
Prednisone mg/day	50±17 (50)
Years of treatment	1.5±0.8 (1.0)
Total FU years	3.0±3.1 (1.7)
ESRD	1

Data are shown as Mean ± SD (Median).

In order to estimate how often sarcoidosis appears as a primary cause of ESRD in our center we reviewed our transplant database and the electronic medical records of the transplant recipients searching for the original renal disease in the native kidney.

From 2000 to the end of 2010, a total of 2,331 patients received a kidney transplant at the Comprehensive Transplant Program at the Johns Hopkins Hospital. The immunosuppressive regimen included induction with either daclizumab or ATG, tacrolimus, mycophenolate, and steroids. Episodes of cellular rejection (both clinical and subclinical) with Banff grades of 1A or 1B were treated with a 3-day pulse of dexamethasone 100 mg/day followed by a taper. If the Banff score was 2A, 2B, or 3 patients received a 7-day course of anti-thymocyte globulin. Both clinical and sub-clinical C4d positive AMR in the presence of DSA was treated with plasmapheresis/IVIg.

A clinical history of systemic sarcoidosis was recorded for 4 kidney transplant recipients. All had inactive disease at the time of transplantation. Two had previous history of lung sarcoidosis, but the cause of ESRD was hypertensive nephrosclerosis. Only two patients (one black female, and one white female) had sarcoidosis in lung and kidney, and lost their native kidneys due to renal sarcoidosis. This suggests that the frequency of sarcoidosis as primary cause of renal failure leading to ESRD could be estimated as less than 1 in 1000 in patients who received a transplanted kidney. Post-transplant, the maintenance immunosuppression may reduce the risk of recurrence of sarcoidosis in transplant recipients.

Review of the transplant outcome in these 4 patients revealed that one lost the graft to chronic changes unrelated to sarcoidosis after 12 years, requiring hemodialysis. Recurrent renal sarcoidosis was detected in a single patient, a 51 year old Caucasian female who had been diagnosed with sarcoidosis at the age of 47, with involvement of the lungs, CNS, and kidney (biopsy-proven) by granulomatous disease, treated with Prednisone 5 mg, and Azathioprine 75 mg daily, and quiescent at the time of transplantation. She received a pre-emptive, HLA-compatible live donor kidney, requiring no pre-transplant desensitization, and with immediate function, resulting in a stable serum creatinine of 1.2 mg/dL (eGFR 48 mL/min/1.73 m^2^). Induction immunosuppression consisted of basiliximab, followed by post-transplant standard maintenance immunosuppressive regimen with Prednisone 5 mg once a day, Tacrolimus 0.5 mg twice a day and Mycophenolate 500 mg twice a day. Recurrence was discovered in an incidental kidney biopsy performed at 17 months post-transplant during a hernia surgical repair, with no evidence of active sarcoidosis, no urinary infections, and stable eGFR 47 mL/min/1.73 m^2^. The biopsy showed tubulointerstitial granulomatous inflammation, with negative C4d, and was interpreted as most consistent with recurrent sarcoidosis, although it was not possible to completely rule out a component of cell mediated rejection (Banff score: g0, i3, t3, v0, ah0, cg0, ci3, ct3, cv1, mm2, ptc undetermined, C4d0, ti3). She was treated for 5 days with Prednisone 80 mg per day, eventually tapered to a maintenance dose of 15 mg per day, while continuing Mycophenolate 500 mg three times per day, and Tacrolimus 1 mg two times per day. The increased immunosuppression was complicated by Herpes virus esophagiitis, and Cytomegalovirus infection, resolved with antiviral treatment, and resolution of infection (eGFR of 34 mL/min/1.73 m^2^ six months later).

## Discussion

The main objectives of this study were to evaluate the incidence of granulomatous tubulointerstitial nephritis in patients with a diagnosis of sarcoidosis; the overall frequency of this diagnosis in native kidney biopsies; the frequency of sarcoidosis as the primary kidney disease leading to ESRD; the incidence of recurrence of renal sarcoidosis in transplanted kidneys.

We found that among patients with a clinical history of sarcoidosis and evidence of renal disease requiring a native kidney biopsy 37% had granulomatous interstitial nephritis.

The demographic characteristics of our cohort of patients with a history of sarcoidosis showed a prevalence of female gender (57%) matching previously observed gender distribution [16], and a significantly higher proportion of black individuals (73%), reflecting estimates that in the US population sarcoidosis is three times more common in blacks (35.5 per 100,000) than in whites (10.0 per 100,000) [2].

Clinical information available on these patients shows that hypercalcemia was present in 7 (21%) of patients with a history of sarcoidosis, similar to previous estimates [17]. However only 3 of these individuals showed sparse microcalcifications in their kidney biopsies.

Granulomatous interstitial nephritis by itself is rare in biopsies from both native kidneys [18], [19], and from transplanted kidneys [20], with an overall estimated occurrence of <1% biopsies. In our center the incidence of this pathologic finding is also very low 0.48% (unpublished observation), consistent with previous reports. In this study, the frequency of granulomatous inflammation ascribable to sarcoidosis in native kidney biopsies appears even lower: 0.18% of 10,023 diagnostic biopsies from native kidney reviewed at our institution.

Given the limited follow up information on the patients with biopsy-proven renal granulomatous sarcoidosis in the native kidney it is not possible to comment on specific characteristics that may influence the course of this renal lesion, except that tubulointerstitial scarring may indicate poor prognosis.

At time of diagnosis serum creatinine was noted to be abnormal in most of these patients, some presenting with acute kidney injury. In some, the possibility that a component of tubulointerstitial damage could be associated with pharmacologic treatment was entertained in the differential diagnosis. Such possibility should be considered in the therapeutic approach in these cases.

It is not clear how many patients with renal sarcoidosis progress to end stage renal disease. In this study, 3 or 5% of a total of 55 patients (including 51 patients with native kidney biopsies and 4 transplant recipients) with a diagnosis of systemic sarcoidosis progressed to ESRD, slightly higher but similar to an estimated 2% reported in a French cohort [21].

Recurrence of renal sarcoidosis post transplantation was documented in only one out of two transplant recipients with ESRD due to sarcoidosis at our center in the past ten years, and was detected 17 months after transplant as an asymptomatic lesion in an incidental biopsy.

Aside from few case reports, the recurrence of sarcoidosis in kidney transplant recipients was examined in a recent, retrospective, multicenter study from France, describing 10 patients with ESRD due to sarcoidosis, who received a kidney transplant [10]. Of these, 3 patients developed granulomatous tubulointerstitial nephritis in the renal graft, representing a 30% recurrence of renal sarcoidosis. The recurrence occurred within 18 months post-transplantation, while the patients were receiving a maintenance immunosuppression regimen including 5 mg prednisone per day. Treatment for recurrence consisted of increasing the corticosteroids dose from 0.3 to 0.5 mg/kg/day for 1 month, followed by a progressive tapering of the dose. The outcome was resolution with improved GFR for one patient, one patient progressed to renal graft failure, and the third patient died from a pulmonary embolism. Perhaps follow-up biopsies at one or two years post transplant may be indicated in to rule out “subclinical” recurrence in transplant recipient with renal granulomatous sarcoidosis as the original disease.

The clinical management and immunosuppression of these rare patients may be challenging. The available literature suggests that steroids should be maintained in the therapeutic regimen post-transplantation to prevent recurrence of disease [7], [8], [9]. Recently, monoclonal antibodies inhibiting interleukin 12, interleukin 23, and tumor necrosis factor alpha have been tested in patients with lung and/or skin sarcoidosis, however, the superiority of this treatment compared with steroids is unclear [22]. The recurrence in our patient was treated with an increased dose of steroids with subsequent decrease of her serum creatinine toward her baseline level. Over-immunosuppression should be avoided to reduce the risk of infection. Over-immunosuppression may have played a role in the lethal fungal infection of the pediatric patient reported by Vargas et al.[9]. The post-biopsy course of our index case suggests that development of CMV and HSV was temporally associated with the escalation in her steroids and other maintenance immunosuppression, and cause and effect relationship seems likely.

In summary, our study indicates that less than 1% of patients with renal abnormalities requiring a native kidney biopsy have a clinical history of sarcoidosis. Our observations on kidney biopsies suggest that in patients with history of sarcoidosis and renal dysfunction about one third shows pathologic manifestation of renal involvement by sarcoidosis as interstitial nephritis with granulomatous inflammation. Although renal involvement by sarcoid granulomatous interstitial nephritis is a rare primary cause of ESRD in transplant recipients, it can recur in the renal allograft.
